# Occurrence of strongyloidiasis in privately owned and sheltered dogs: clinical presentation and treatment outcome

**DOI:** 10.1186/s13071-017-2275-5

**Published:** 2017-07-20

**Authors:** Paola Paradies, Fabrizio Iarussi, Mariateresa Sasanelli, Antonio Capogna, Riccardo Paolo Lia, Daniele Zucca, Beatrice Greco, Cinzia Cantacessi, Domenico Otranto

**Affiliations:** 1 Department of Emergency and Organ Transplantations, Veterinary Section, University of Bari, 70010 Valenzano, Bari Italy; 2 Department of Veterinary Medicine, University of Bari, 70010 Valenzano, Bari Italy; 3 Institute of Animal Health, University of Las Palmas de Gran Canaria, Las Palmas, Spain; 4 Department of Veterinary Medicine, University of Cambridge, Cambridge, UK

**Keywords:** *Strongyloides stercoralis*, Zoonosis, Treatment, Faecal monitoring, Dog, Clinical presentation

## Abstract

**Background:**

The increasing number of reports of human infections by *Strongyloides stercoralis* from a range of European countries over the last 20 years has spurred the interest of the scientific community towards this parasite and, in particular, towards the role that infections of canine hosts may play in the epidemiology of human disease. Data on the epidemiology of canine strongyloidiasis is currently limited, most likely because of the inherent limitations of current diagnostic methods.

**Methods:**

Faecal samples were collected directly from the rectal ampulla of 272 animals of varying age and both genders living in Apulia, southern Italy. Dogs included were either privately owned (*n* = 210), living in an urban area but with unrestricted outdoor access (Group 1), or shelter dogs (*n* = 62 out of ~400) hosted in a single shelter in the province of Bari in which a history of diarrhoea, weight loss, reduced appetite and respiratory symptoms had been reported (Group 2). *Strongyloides stercoralis* infection was diagnosed by coproscopy on direct faecal smear and via the Baermann method.

**Results:**

Six of 272 dogs were positive for *S. stercoralis* at the Baermann examination; all but one were from the shelter (Group 2) and displayed gastrointestinal clinical signs. The only owned dog (Group 1) infected with *S. stercoralis*, but clinically healthy, had been adopted from a shelter 1 year prior to sampling. Five infected dogs were treated with fenbendazole (Panacur®, Intervet, Animal Health, 50 mg/kg, PO daily for 5 days), or with a combination of fenbendazole and moxidectin plus imidacloprid spot-on (Im/Mox; Advocate® spot-on, Bayer). Post-treatment clearance of infection was confirmed in three dogs by Baermann examination, whereas treatment failure was documented in two dogs by Baermann and/or post-mortem detection of adult parasites.

**Conclusions:**

This study describes, for the first time, the presence of *S. stercoralis* infection in sheltered dogs from southern Italy. Data indicate that *S. stercoralis* infection may pose a concern for sheltered animals and raise questions on potential risks of infection for staff of municipal shelters in southern European countries. Given that a single course of treatment with fenbendazole, associated or not with Im/Mox spot-on, may not eliminate the infection, effective treatment protocols should be investigated and control strategies targeting the environment considered for reducing the risk of zoonotic infection.

## Background

*Strongyloides stercoralis* (Rhabditida: Strongyloididae) is the causative agent of strongyloidiasis in a range of vertebrate hosts, including humans and dogs [1], particularly in tropical and subtropical areas of the world (e.g. Africa, South America). The life-cycle of *S. stercoralis* is peculiar, in that it includes sexual reproduction and multiplication by parthenogenesis (reviewed in [2]). In particular, adult parasitic females in the vertebrate hosts [3] reproduce via parthenogenesis and produce both male and female offspring. While the former will develop (via moult through four larval stages) into free-living adult nematodes, the latter develop through to third stage-larvae (L3s), which can either complete their development to free-living females or infect a vertebrate host (reviewed in [2]). Importantly, the offspring derived from the sexual reproduction of free-living males and females is inevitably parasitic [2]. Parasitic larvae mainly penetrate the skin and mucosal tissues of vertebrate hosts, although lactogenic transmission has also been experimentally demonstrated in dogs [4]. Another described route of infection (in both humans and dogs) involves autoinfection by first-stage larvae (L1), which subsequently develop through to infective L3s within the intestinal mucosa and/or in the perianal region of the host [5, 6].

In immunocompetent individuals, the disease is mostly asymptomatic, whereas in immunocompromised subjects the parasites can disseminate to visceral organs and tissues, a condition known as ‘disseminated strongyloidiasis’ [7]. Similarly, in dogs, clinical signs of strongyloidiasis include asymptomatic to severe conditions, characterized by dermatological, gastrointestinal and/or respiratory signs, mostly in young animals [1]. Interestingly, while data on the prevalence of *Strongyloides* infections in dogs in Asia and South America are available [8, 9]), with some areas considered endemic for this parasite [10, 11], little is known about the presence of this parasite in dogs in Europe, with published records limited to single foci of infection (e.g. Germany [12], Finland [5], Greece [13] and France [14]). However, the increasing number of reports of human infections by *S. stercoralis* recorded in a range of European countries over the last 20 years [15] has spurred the interest of the scientific community towards this parasite. In particular, the role that infection of domestic dogs may play in the epidemiology of the human disease is still under debate [16]. Despite the increased attention, data on the epidemiology of canine strongyloidiasis are currently limited, most likely as a consequence of the intrinsic limitations in the diagnostic techniques currently used for the detection of infections in dogs. Indeed, although serological tests (IFAT and ELISA) have been developed for this purpose [1], detection of parasites in faecal samples using the Baermann technique on faecal samples remains widespread. While this is often considered the “gold standard” in clinical practice and diagnostic laboratories, sensitivity is limited, mainly because of the small amount of faeces used and the intermittent shedding of first-stage larvae (L1s). Therefore, multiple samplings are required to unequivocally rule out the presence of larvae in the faecal matter [6, 17].

The acquisition of data on the prevalence of infection in canine populations in Europe is crucial to assess the real risk of zoonotic transmission to humans. In addition, knowledge of the range of clinical signs associated with the presence of *S. stercoralis* in dogs is essential in order to ensure that infection by this parasite is inserted amongst the list of differential diagnoses in animals presented with compatible clinical signs. Given the routes of transmission to dogs, we hypothesize that animals with unrestricted outdoor access are likely to be continuously exposed to the infection, with dogs in shelters being significantly more likely to acquire the parasite when compared with animals kept as human companions. However, thus far, no study has investigated the difference in prevalence of *S. stercoralis* infection in dog cohorts coming from the same geographical area but characterised by different lifestyles. In the present study, we filled this gap in knowledge by investigating the occurrence of canine strongyloidiasis in owned dogs from an urban area and sheltered dogs, and describe clinical and pathological features of the infection in six dogs along with clinical presentations and treatment outcomes.

## Methods

### Study design

Faecal samples were collected directly from the rectal ampullae of 272 animals of varying age and both genders (see Table 1) living in Apulia, southern Italy. Dogs of all ages and breeds were enrolled in the study; anamnestic data including dog history, living conditions (e.g. house or apartment with outdoor access, shelter), location, instances of travel to other regions or abroad as well as medical history, were collected and registered on individual clinical forms when possible. All clinical procedures described below were part of routine clinical care. In particular, dogs from 2 groups were enrolled:

**Table 1:** Number and percentage of dogs from Groups 1 (privately owned) and 2 (shelter) enrolled in the study listed according to gender, breed, age and occurrence of clinical signs potentially suggestive of strongyloidiasis (i.e. gastrointestinal and/or respiratory signs)

		Group 1		Group 2	
		Total	%	Total	%
Dogs		210		62	
Gender	Male	116	55	25	40
	Female	94	45	37	60
Age	Young < 2 yrs	46	22	13	21
	Adult 2–7 yrs	81	39	27	44
	Senior > 7 yrs	83	40	22	35
Breed	Pure breed	84	40	0	
	Cross breed	126	60	62	100
Clinical status	Gastrointestinal or respiratory signs	42	20	22	35
	Healthy or other signs	168	80	40	65

Group 1: privately owned dogs (*n* = 210) living in an urban area but with unrestricted outdoor access. These dogs were presented with a variety of different clinical conditions (including gastrointestinal signs and/or respiratory signs, *n* = 42) or for routine clinical examinations at the Clinical Unit of the Veterinary Teaching Hospital of the University of Bari.

Group 2: shelter dogs (*n* = 62) in the Province of Bari (41°04′47″N, 16°55′17″E). In the shelter, despite an ongoing anthelmintic treatment program and controlled alimentary regime, a history of diarrhoea, weight loss and reduced appetite of unknown origin was reported in some of the dogs over the previous years. Animals sampled were either dogs that displayed clinical signs (*n* = 22) (i.e. diarrhoea, weight loss, reduced appetite, respiratory signs) or healthy dogs (*n* = 40) located in pens in proximity of dogs with clinical signs (see above). Animals were housed in wire mesh cages (approximately 10 × 20 m), 4 to 7 animals per pen, according to their gender, and existing hierarchies within each group. The pens were made of concrete flooring and were cleaned with jet water twice a day.

### Diagnostic procedures

*Strongyloides stercoralis* infection was diagnosed by coproscopy on direct faecal smear and/or via the Baermann method [18]. Any recovered L1s were identified according to morphological keys [19]. In case of death of the animal (see below), necropsy was performed for parasitological and histopathological examination. Small portions of the duodenum, jejunum and colon were recovered and immediately scraped and washed for parasite detection. Parasites were clarified in 20% lactophenol and examined under the optical microscope. Adult females were processed for scanning electron microscopy (SEM). Samples from different regions of the gut and from all the major organs (kidneys, liver, spleen, lungs) were collected to verify larval dissemination and fixed in 10% buffered formalin for histopathological processing; samples were processed, embedded in paraffin, sliced at 4 μm and stained with haematoxylin and eosin.

### Clinical presentation and follow-up of positive dogs

Out of the six animals positive to *S. stercoralis*, five (dogs 1–5) were presented with clinical alterations compatible with strongyloidiasis (i.e. severe gastrointestinal disease associated with hypoproteinemia). These animals, all from Group 2, had a history of chronic disease (weight loss, diarrhoea, reduced appetite, vomiting) except for Dog 3 that was presented with a hyper-acute onset of depression, anorexia and vomiting that had begun 2 days prior to sampling. One week prior to the enrolment, the clinical conditions of Dogs 4 and 5 had worsened, with acute vomiting and severe watery diarrhoea, respectively. Haematological and biochemical analysis, including C reactive protein and serum protein electrophoresis, were performed in all animals. Flotation faecal test and ELISA for *Giardia* spp. antigens were performed to exclude concomitant parasitic infections. Supportive therapy was administered when needed, together with metronidazole (10 mg/kg, PO bid) to control intestinal bacterial overgrowth in dogs with diarrhoea. Clinical and parasitological monitoring of *Strongyloides*-positive dogs was performed daily by Baermann examination of faecal samples until the first negative result (first follow-up), and subsequently repeated twice a month (Table 2), on a three-day pooled faecal sample.

**Table 2:** Results of faecal monitoring. Presence/absence of *Strongyloides stercoralis* motile larvae on 3 days faecal pools collected directly from the dog ampullae

Faecal monitoring		Dog 2	Dog 3	Dog 4	Dog 5	Dog 6
Diagnosis/Treatment start	D0	Positive	Positive	Positive	Positive	Positive
Follow-up 1		Negative (D 7, 8, 9)	Negative (D 6, 7, 8)	Negative (D 7, 8, 9)	Negative (D 10, 11, 12)	Negative (D 6, 7, 8)
Follow-up 2		Negative (D 24, 25, 26)	Negative (D 23, 24, 25)	Negative (D 24, 25, 26)	Negative (D 27, 28, 29)	na
Follow-up 3		Positive (D 41, 42, 43)	Negative (D 40, 41, 42)	Negative (D 41, 42, 43)	Negative (D 44, 45, 46)	na
Follow-up 4		na	Negative (D 57, 58, 59)	Negative (D 58, 59, 60)	na	na

*Abbreviations*: *D* days post-treatment, *na* not available

## Results

**Fig. 1:**
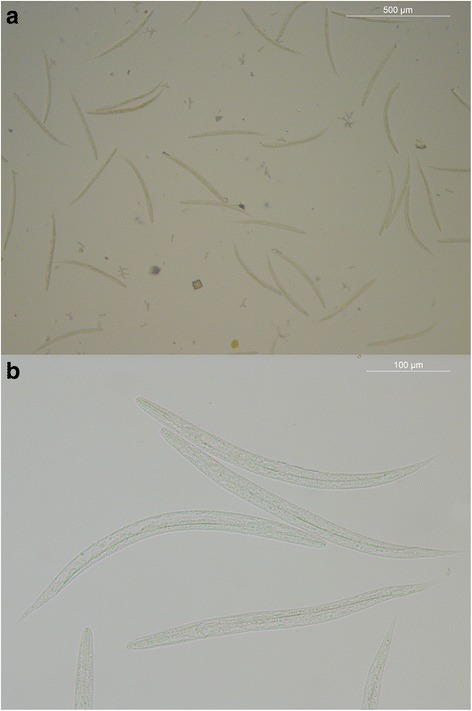
**a**, **b** *Strongyloides stercoralis* L1 rhabditiform larvae observed in fresh faecal smear following clarification in 20% lactophenol. *Scale-bars*: **a**, 500 μm; **b**, 100 μm

**Fig. 2:**
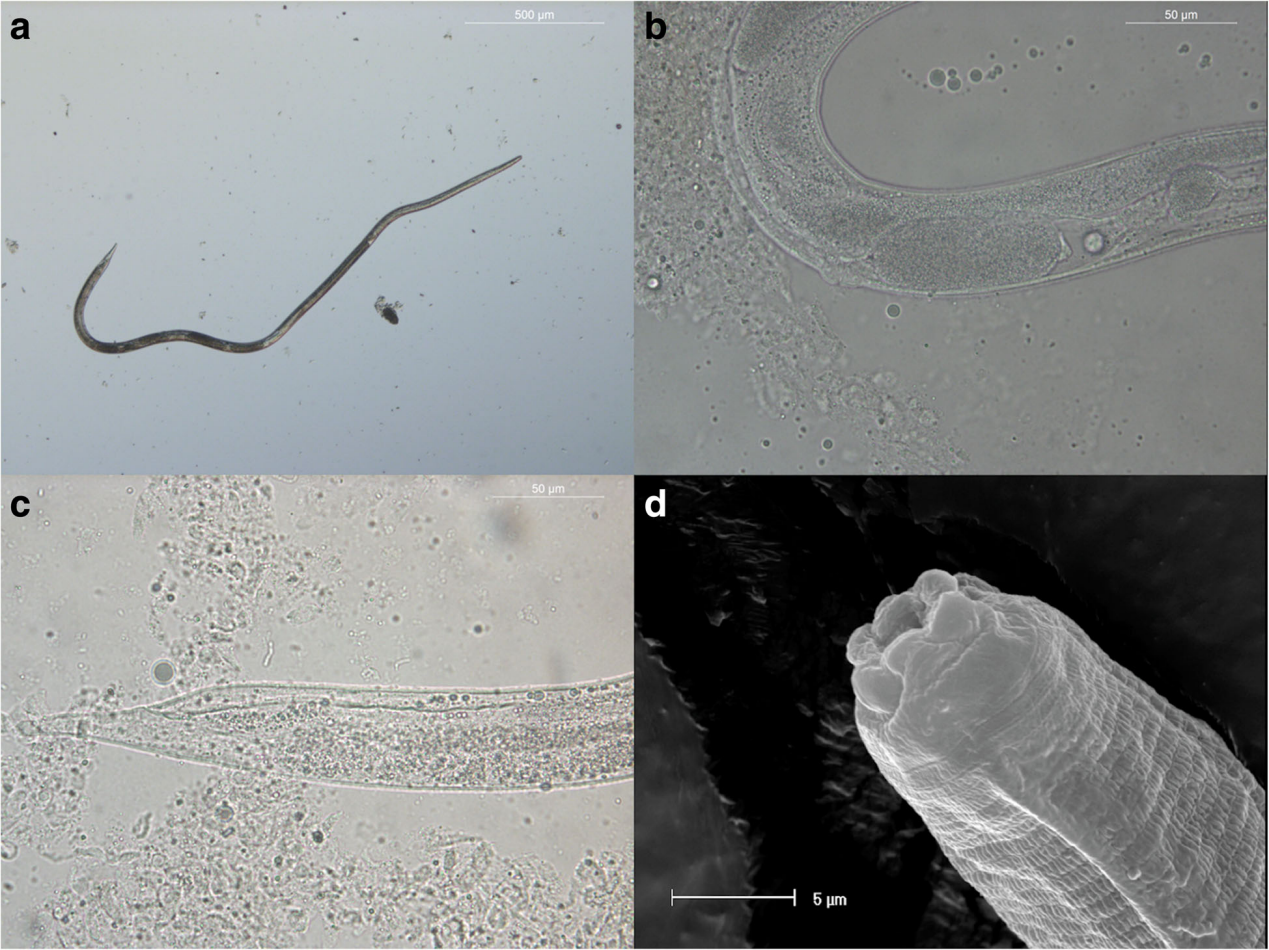
Adult *Strongyloides stercoralis* female collected from a duodenal scraping. **a** Parasitic female in toto: oesophagus length is appreciable. **b** Position of the vulva and intrauterine eggs. **c** Narrowly tapered tail. **d** Cephalic region observed under SEM. *Scale-bars*: **a**, 500 μm; **b**, 50 μm; **c**, 50 μm; **d**, 5 μm

**Fig. 3:**
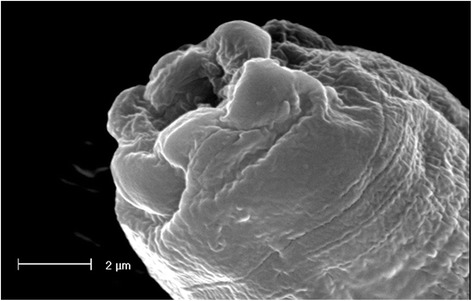
Scanning electron micrograph of the cephalic region of *Strongyloides stercoralis* adult female; note the exagonal shape of the mouth. *Scale-bar*: 2 μm

**Fig. 4:**
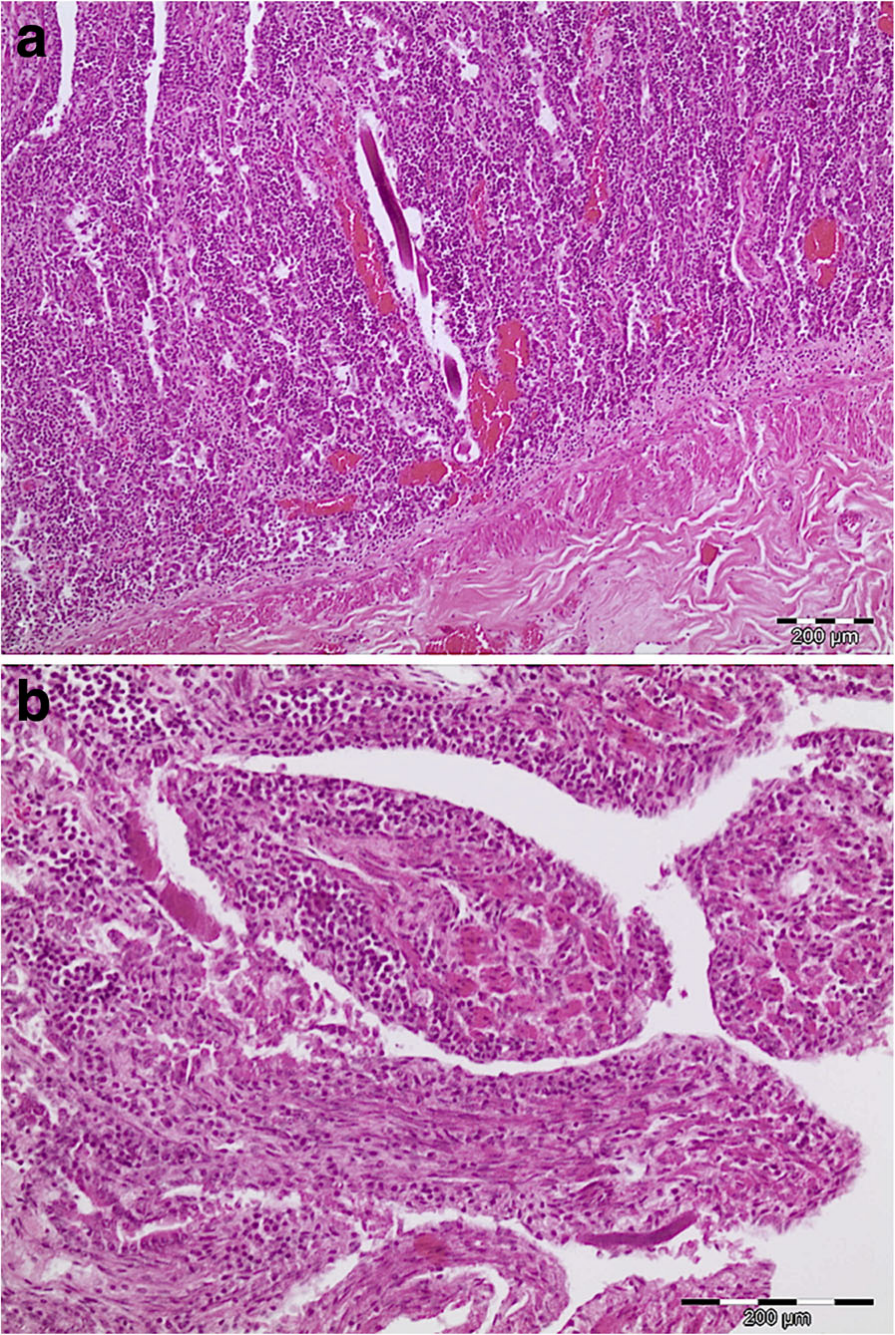
Histopathology: duodenum (hematoxylin and eosin staining). **a** adult *Strongyloides stercoralis* in the mucosa (magnification of 10 × 10). **b** Eggs, larvae and fragments of adults in the mucosa (magnification of 10 × 20)

One privately owned dog (0.5%, 1/210; Group 1), clinically healthy, and five shelter dogs (8.1%, 5/62; Group 2) displaying gastrointestinal clinical signs, were positive for *S. stercoralis* (Table 3 ). The only owned dog (Group 1) scoring positive had been adopted from a different shelter 1 year prior to sampling. The clinical and pathological abnormalities observed in the positive dogs are reported in Table 3. All symptomatic positive dogs were thin (body condition score- BCS 2-4) and dehydrated, except for Dog 3. Dog 1 was cachectic, highly depressed, hypothermic, severely dehydrated and died within 24 h from admission. Dog 2 showed an abnormal mass at abdominal palpation, which was later diagnosed as type B intestinal lymphoma. Abdominal palpation in Dog 4 evoked pain. Diarrhoea was observed in three out of five *Strongyloides*-positive dogs. Respiratory signs were not observed in any of the dogs. The most frequent laboratory changes were mild anemia with hypoalbuminemia (5/5) and leucocytosis, neutrophilia, panhypoproteinemia and increased CRP (4/5). Mild eosinophilia was observed only in one case (Dog 4). Furthermore, serum protein electrophoresis showed a variable increase in α2- globulin fraction in all *Strongyloides*-positive dogs. For Dog 3, the diagnosis was achieved belatedly during hospitalization, as the faecal sample collected at presentation was negative. Dogs 2, 3 and 6 were treated with fenbendazole (Panacur®, Intervet, Animal Health, 50 mg/kg, PO daily for 5 days), whereas Dogs 4 and 5 with a combination of fenbendazole and moxidectin plus imidacloprid spot-on (Im/Mox; Advocate® spot-on, Bayer). No side effects were recorded following treatment and positive dogs remained in the hospital until the end of the monitoring period to prevent reinfection. The results of coproscopy at follow-up are reported in Table 2. Briefly, the first negative results were observed from 6 to 12 days following administration of treatment, and confirmed on three-day pooled faecal samples. Dog 3, 4 and 5 remained negative for the parasite until the last follow-up. Dog 6 was negative at the first and only follow-up available. In Dog 2, larvae were not detected at the first and second follow-up, while the faecal sample collected at the third follow-up was again positive for the parasite. Furthermore, this dog experienced adverse reactions to the lymphoma chemotherapy protocol and was therefore euthanized with the consent of the shelter manager. Dog 3 improved quickly following fenbendazole treatment (within 1 week), whereas Dog 4 improved slowly, reaching a normal clinical status only at the end of the monitoring period. Despite faecal consistency gradually improving following treatment, Dog 5 died as a consequence of the severe ongoing protein loosing enteropathy after the third follow-up. The scraping of the intestinal mucosa of Dog 1 (left untreated and deceased within 24 h from admission) showed a high parasitic burden. Larval stages were particularly abundant and L1s detected in the faeces (200–300 μm in length) presented a typical rhabditiform shaped esophagus and a prominent genital primordium (Fig. 1). Adult nematodes (Fig. 2), recovered from the intestinal mucosa of the duodenum only, were females (2.0–2.5 mm in length) presenting a long cylindrical oesophagus, the vulva located in the posterior third of the body, a narrowly tapered tail and a genital tract paired with the uteri filled with a small number of developing eggs. SEM allowed visualisation of the cephalic region of the *S. stercoralis* adult female, with its hexagonal-shaped mouth (Fig. 3). In Dogs 2 and 5, scraping of the intestinal mucosa revealed the presence of few different parasitic stages and only rare adult females, respectively. Histopathological examination of Dog 1 revealed severe hemorrhagic lymphoplasmacellular enteritis involving the entire intestinal tract. Adult nematodes, larvae and eggs (Fig. 4) were observed in the duodenum. Furthermore, moderate interstitial pneumonia and mild atelectasis were observed. In Dogs 2 and 5, a mild and moderate inflammatory infiltrate in the duodenum, mainly consisting in lymphocytes and plasma cells, respectively, were the only pathological finding reported. Migrating larvae could not be detected in viscera of any of the dogs.

**Table 3:** Clinical signs and results of selected laboratory parameters at presentation (D0) in the six *Strongyloides stercoralis*-infected dogs

	Normal range	Dog 1	Dog 2	Dog 3	Dog 4	Dog 5	Dog 6
History and clinical presentation		Long-lasting diarrhea and weight loss; cachexia; anorexia; depression; hypothermia	Weight loss; reduced appetite; abdominal mass	Acute onset of depression; anorexia and vomiting	Chronic weight loss and episodic diarrhoea; acute vomiting and anorexia	Chronic weight loss and episodic diarrhoea; severe watery diarrhoea of 1 week duration	Adopted from the shelter 1 year before; healthy
WBC (k/μl)	6.00–17.00	32.6	29.9	24.7	17.00	7.0	8.3
NEU (%)	60–77	90	86	86	80	77	67
LYM (%)	12–30	2	3	5	5	10	17
EOS (%)	2–10	4	3	4	12	8	8
RBC (M/μl)	5.5–8.5	5.2	4.53	5.5	5.5	5.2	5.6
HGB (g/dl)	12–18	10.6	9.3	11.6	11	11.4	11.8
Hct (%)	37–55	28.5	28.1	34	35.1	34.7	36
Tot. Prot. (g/dl)	5.5–7.8	3.2 (Alb 1.0)	5.8 (Alb 1.3)	4.8 (Alb 2.4)	3.6 (Alb 1.1)	3.6 (Alb 1.2)	5.6 (Alb 2.5)

*Note*: CRP (C reactive protein); PLT (platelet) and MONO% (% monocites) were all within normal range

## Discussion

This study documents, for the first time, the presence of *S. stercoralis* infection in sheltered dogs from southern Italy. Given the limited sensitivity of the current diagnostic techniques used to detect the infection, the prevalence of this parasite in canine populations (especially in shelters, where the risk of transmission is higher), is most likely underestimated. In Italy, the occurrence of *S. stercoralis* in canine faecal samples had been diagnosed in northern [16] and central regions [20], while a single case report has been described in a national journal [21]. *Strongyloides stercoralis* infections are often asymptomatic in dogs; this, together with the low reliability of the Baermann sedimentation as a gold diagnostic standard, makes the assessment of the ‘true’ prevalence of infection challenging. The limited sensitivity of this test was clearly indicated by the fact that, in Dog 5, no larvae could be detected in the faecal samples collected at the post-treatment follow-ups, whereas intestinal mucosal scraping revealed the presence of rare adult females (Dog 5). In addition, in Dog 3, diagnosis was achieved following repeated testing, likely as a consequence of low larval counts in the faecal sample or to intermittent larval shedding, often occurring in chronic infections [1]. In humans, false negative results could translate into crucial (and potentially fatal) misdiagnoses, as undetected infections may leave the patient exposed to the risk of developing disseminated strongyloidiasis at any time in life [15]. Accordingly, serological testing has been proposed to monitor the efficacy of anthelmintic treatment in human strongyloidiasis [22]; such tests (or similar assays, including molecular-based) would be invaluable for use towards the diagnosis of canine strongyloidiasis in order to prevent the occurrence of false negative results.

A thorough knowledge of the range of clinical presentations of canine strongyloidiasis, together with the biochemical alterations in affected dogs, is crucial in order to consider inclusion of this infection amongst the list of differential diagnoses in dogs with compatible signs. While in dogs, symptomatic strongyloidiasis is often associated with young animals or puppies [5, 23], in this study we have reported the occurrence of cases of symptomatic *S. stercoralis* infection in adult dogs, with or without concomitant infections. These were characterised by severe clinical signs, with three deaths observed; however, based solely on our observations, neither of these deaths could be ascribed to *Strongyloides* dissemination. Indeed, Dog 1 and Dog 5 died most likely as a result of long lasting intestinal damage, and screening of visceral organs and tissues did not reveal the presence of larvae. On the other hand, while the exact cause of death of Dog 2 could not be established, the presence of a concomitant tumour that did not respond to administration of chemotherapeutics is likely to have contributed significantly to this outcome, similarly to previous observations in humans [24]. Therefore, based on the results in our study, the occurrence of disseminated strongyloidiasis in dogs [25] could not be established. Although none of the laboratory changes shared by the *Strongyloides*-positive dogs (e.g. leukocytosis with neutrophilia, mild anemia, hypoalbuminemia, increase in CRP and α2 globulin fraction) is specific for the infection, the combination of these findings could increase the suspicion of the disease. Conversely, eosinophilia, which is often associated with human strongyloidiasis [26], was observed only in one dog.

Together with the low sensitivity of the diagnostic tests currently used and the scarce information on the clinical presentation of the disease, detection of canine strongyloidiasis is also impaired by the challenges in differentiating *S. stercoralis* larvae from larvae of free-living nematodes, which may be present in faecal samples. Therefore, the direct collection of faecal samples from the rectal ampulla rather than from the environment, and a thorough morphological discrimination of larvae shed by other parasitic nematodes of dogs (i.e. *Angiostrongylus vasorum*, *Crenosoma vulpis*, *Oslerus osleri*, *Filaroides hirti* and *Filaroides milksi*) is warranted, and contributes to provide a more reliable snapshot of the distribution of this infection in canine populations.

The efficacy of a single course of treatment with fenbendazole, associated or not with Im/Mox spot-on was not 100% effective in eliminating the infection. Fenbendazole was selected based on its safety record, as well as based on its administration route, while the association of Im/Mox was intended to increase the effectiveness against the infection. On the whole, data on the efficacy of treatments against canine strongyloidiasis is limited to two studies [27, 28] and single case reports [29] or anectodal experiences. Fenbendazole was previously shown to be effective against *Strongyloides* in six naturally infected dogs (out of seven) from Japan [27]. Treatment with ivermectin, i.e. the treatment of choice in human strongyloidiasis [30], had been previously tested in three experimentally- and two naturally infected dogs [28]. One of the latter dogs suffered a recrudescence, while treatment of experimentally infected dogs was not effective in clearing third-stage larvae from parenteral sites [28]. In our study, the efficacy of a single course of treatment with fenbendazole, associated or not with Im/Mox spot-on did not consistently result in the elimination of the infection. It could be argued that this outcome is related to the severity of clinical signs observed at admission and that a favourable response (i.e. no larval excretion) may still be seen in other cases, thus highlighting the importance of a prompt diagnosis. Furthermore, because of the abovementioned limitations of the Baermann technique, faecal monitoring by molecular tools is likely to represent a better choice when evaluating the efficacy of treatment in individual dogs.

Overall, our data indicate that *S. stercoralis* infection may represent a concern for sheltered animals and point to the potential risk of infection for personnel working in the large number of municipal shelters [31] present in southern European Countries (e.g. Italy, Spain, Portugal, Greece). Indeed, limited financial resources in such contexts may impair the implementation of regular deworming programs, thus increasing the risk of zoonotic transmission of this infection. All infected dogs described in this study were housed in the shelter and were neutered, thus indicating that lactogenic transmission did not play a role in the maintenance of the infection in the shelter (cf. [5]), and that this was spread via contaminated faeces. Under these circumstances, the application of correct deworming protocols [28] is necessary to reduce the environmental infective larval burden and, therefore, protect dogs and workers alike from the risk of infection.

## Conclusions

The study describes the occurrence of *S. stercoralis* infection in shelter dogs from Italy. Based on our observations, we advocate for an increase awareness of this disease, for both owners and veterinarians, and of its potential zoonotic risk. Therefore, we propose that infection by *S. stercoralis* should be included in the list of differential diagnoses of gastrointestinal disease. In addition, considering that a single course of treatment with fenbendazole, associated or not to Im/Mox spot-on may be ineffective to eliminate the infection, control strategies targeting the environment should be implemented to reduce the risk of infection. Importantly, monitoring programs managed by health authorities are necessary to limit the impact of this disease on human and canine populations alike.

## Abbreviation

CRP: C reactive protein
